# Exposure to a Fungal Volatile Compound: Significance of Effects

**DOI:** 10.1289/ehp.114-a338a

**Published:** 2006-06

**Authors:** Andrew Saxon

**Affiliations:** Department of Medicine, UCLA Medical School, Los Angeles, California, E-mail: asaxon@mednet.ucla.edu

In contrast to what Wålinder et al. (2005) concluded in their article “Acute Effects of a Fungal Volatile Compound,” I interpret the article to report essentially no effects beyond chance. In all, the authors carried out some 76 comparisons (each one representing a time point and an exposure vs. control measurement) if you take blink frequency as a single comparison. The authors reported finding 5 “significant differences” out of 76 comparisons. Of the reported significant differences, one (blink frequency) is misleading, as discussed below. Of the remaining 75 comparisons, 4 differences at a *p*-value of < 0.05 might be expected by random chance. This is without applying Bonferroni’s adjustment for multiple comparisons; using this adjustment, a *p*-value of approximately < 0.0007 would be required for a single comparison to be statistically significant. None of the differences reported reached this level.

Wålinder et al. (2005) reported that the subjects showed increased “blink frequency” during 3-methylfuran (3-MF) exposure (Table 1), but the frequency was higher in the exposure phase at time 0, about 9 for 3-MF exposure versus 6.5 for the control air phase. From the data in Table 1, it appears that both groups had fewer overall blinks per minute compared with baseline during the trial (Figure 2). Reporting that blinking was higher during exposure and not noting that it was higher at baseline is disingenuous.

Wålinder et al. (2005) may have mis-labeled tear break-up, but as it reads in the legend for Table 1, a negative value indicates a decrease; therefore, the 6 sec given for measured break-up time after 3-MF exposure (Table 1) indicates that it was increased (i.e., longer to tear break-up), which is better. Is this correct? Also, was the observer who measured the tear break-up blinded to the exposure?

Finally, of the four lung measurements taken, the only comparison with a *p*-value of < 0.05 was the small 100-mL change for forced vital capacity (FVC) right after exposure (Table 4). How do the authors interpret this change in FVC in view of the fact that there was no significant change in forced expiratory volume in 1 sec (FEV_1_)?
